# A Global Challenge: Clean Drinking Water

**DOI:** 10.1002/gch2.202000125

**Published:** 2021-01-07

**Authors:** Xiuqiang Li, Hui Ying Yang

**Affiliations:** ^1^ Department of Mechanical Engineering and Material Science Duke University Durham NC 27708 USA; ^2^ Engineering Product Development Pillar Singapore University of Technology and Design Singapore 487372 Singapore

Water is an essential element for life. A minimum of 5L/day of drinking water is required for a person to survive with normal activities.^[^ ^1^ ^]^ Currently, 2.1 billion people globally have limited access to safe drinking water. Additionally, about 6% of deaths in underdeveloped countries are caused by drinking unsafe water.^[^ ^2^ ^]^ To address water scarcity, the research community has proposed a variety of solutions to alleviate the drinking water crisis, such as solar desalination, reverse osmosis (RO), atmospheric water harvesting, and capacitive deionization technology (CDI). However, the high energy consumption, low efficiency and/or high production cost of these technologies greatly hinder the future development of these devices. However, with the development of material science and nanotechnology, some profound changes are taking places in these technologies. This special issue features four review papers and three research papers that focus on solar desalination, atmospheric water harvesting and CDI for clean drinking water, which aim to further promote the development of related technologies.

Direct solar desalination, which produces freshwater directly using solar energy with minimum carbon footprint, is considered to be one of the most promising technologies to alleviate the water shortage crisis. However, the traditional bulk water heating method is inefficient (≈40%). Recently, interfacial solar vapor/steam generation has been proposed to improve heat localization at the liquid surface and has achieved ≈90% solar‐to‐vapor conversion efficiency under 1 Sun. In this special issue, a review paper contributed by Irshad et al. (article number 2000055) systematically summarizes the progress of solar vapor/steam generation and constructively points out the key directions of future research. As mentioned in this review, the long‐term stability of absorbers is still a challenging issue. Zhuang et al. (article number 2000053) and Gu et al. (article number 2000063) demonstrate two advanced materials, reduced graphene oxide hydrogel membranes and three‐dimensional honeycomb chitosan‐based aerogels, which exhibit long‐term stability without compromising the water evaporation rate (> 1.7 kg m^−2^ h^−1^). These represent competitive materials for salt‐resistant absorbers. Meanwhile, an impressive work hoping to thoroughly solve the problem of salt‐rejecting of absorbers is presented by Bian et al. (article number 2000077) who select a highly efficient selective absorber and use convection between the selective absorber and water to heat the water (the absorber was suspended above the water instead of traditional direct contact). The results show that the evaporation efficiency is able to reach 1.94 kg m^−2^ h^−1^. Additionally, it is worth noting that the advantages of the regulation and utilization of infrared light have been reported recently, with the use of selective absorbers to obtain higher efficiency, and the use of radiative cooling to enhance condensation, etc., becoming a key focus for researchers. In this special issue, Li et al. (article number 2000058) offer a fundamental understanding of spectrum design, alongside a discussion of recent progress and future directions of for this research.

Atmospheric water harvesting, which captures the moisture from air and then condenses the captured moisture into liquid water, is a promising technology to resolve the water crisis in arid regions. Compared with traditional inorganic salts, zeolites, etc., recently developed materials, such as MOFs/COFs and hydrogels, are demonstrated to have lower desorption energy and/or humidity adsorption. In this special issue, Zhuang et al. (article number 2000085) contribute a review paper to discuss and provide a perspective regarding various atmospheric water harvesting technologies, especially for solar atmospheric water harvesting with the above‐mentioned advanced materials. In addition, CDI technology is also a competitive technology that uses electrode materials to extract positive and negative ions in the feed solution to achieve water purification. Liu et al. (article number 2000054) review various sodium‐ion intercalation materials as highly efficient CDI electrodes, and provide some instructive perspectives. We believe that these papers will be helpful for both the research and industrial community to achieve new milestones and to shape future research directions.

As United Nations Development Programme 6 mentions,^[^ ^3^ ^]^ with the increase of drought and desertification, more and more countries will suffer from water shortages. By 2050, it is predicted that at least one in four people will suffer from recurring water shortages. Herein, learning from the perspectives of the authors in this special issue, we believe that more efficient devices based on new mechanisms, new materials, and new structures should be developed. In addition, the stability and price of the devices need to be considered in the long‐term research and industrial plan. The community should also actively communicate and collaborate with industry and the public to apply these advanced technologies into practice to alleviate the drinking water challenges of the future.
